# Enhancing Robustness of Adhesive Hydrogels through PEG-NHS Incorporation

**DOI:** 10.1021/acsami.3c13062

**Published:** 2023-10-23

**Authors:** Ece Uslu, Vijay Kumar Rana, Yanheng Guo, Theofanis Stampoultzis, François Gorostidi, Kishore Sandu, Dominique P. Pioletti

**Affiliations:** † Laboratory of Biomechanical Orthopaedics, Institute of Bioengineering, School of Engineering, EPFL, Lausanne 1015, Switzerland; ‡ Airway Sector, Médecine Hautement Spécialisée, Department of Otorhinolaryngology, University Hospital CHUV, Lausanne 1011, Switzerland

**Keywords:** Adhesive Hydrogels, PEG-NHS, Durability, Tracheomalacia, *Ex Vivo*

## Abstract

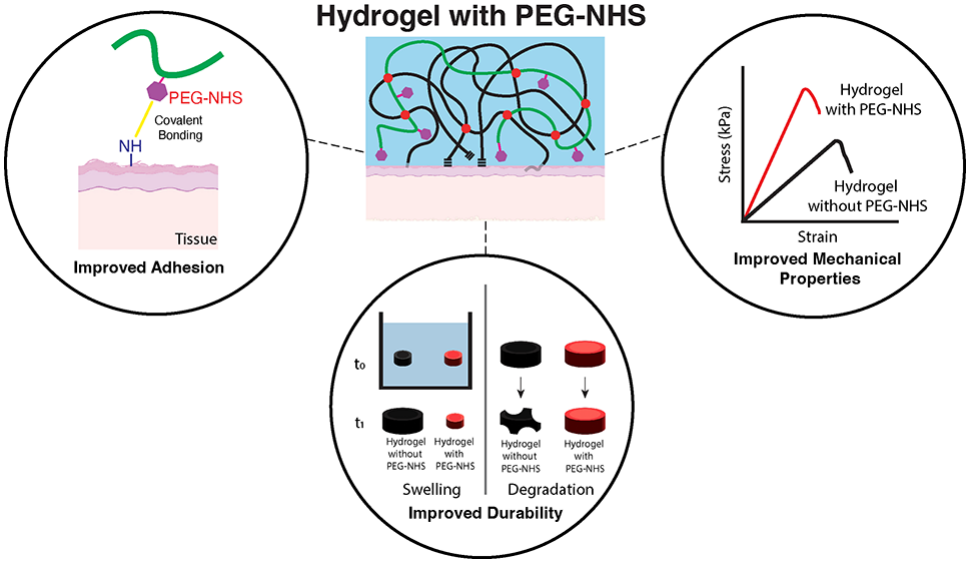

Tissue wounds are a significant challenge for the healthcare system, affecting millions globally. Current methods like suturing and stapling have limitations as they inadequately cover the wound, fail to prevent fluid leakage, and increase the risk of infection. Effective solutions for diverse wound conditions are still lacking. Adhesive hydrogels, on the other hand, can be a potential alternative for wound care. They offer benefits such as firm sealing without leakage, easy and rapid application, and the provision of mechanical support and flexibility. However, the *in vivo* durability of hydrogels is often compromised by excessive swelling and unforeseen degradation, which limits their widespread use. In this study, we addressed the durability issues of the adhesive hydrogels by incorporating acrylamide polyethylene glycol *N*-hydroxysuccinimide (PEG-NHS) moieties (max. 2 wt %) into hydrogels based on hydroxy ethyl acrylamide (HEAam). The results showed that the addition of PEG-NHS significantly enhanced the adhesion performance, achieving up to 2-fold improvement on various soft tissues including skin, trachea, heart, lung, liver, and kidney. We further observed that the addition of PEG-NHS into the adhesive hydrogel network improved their intrinsic mechanical properties. The tensile modulus of these hydrogels increased up to 5-fold, while the swelling ratio decreased up to 2-fold in various media. These hydrogels also exhibited improved durability under the enzymatic and oxidative biodegradation induced conditions without causing any toxicity to the cells. To evaluate its potential for clinical applications, we used PEG-NHS based hydrogels to address tracheomalacia, a condition characterized by inadequate mechanical support of the airway due to weak/malacic cartilage rings. *Ex vivo* study confirmed that the addition of PEG-NHS to the hydrogel network prevented approximately 90% of airway collapse compared to the case without PEG-NHS. Overall, this study offers a promising approach to enhance the durability of adhesive hydrogels by the addition of PEG-NHS, thereby improving their overall performances for various biomedical applications.

## Introduction

Tissue wounds range from minor skin cuts to serious injuries, affecting millions worldwide.^1,2^ In the USA alone, tissue wounds affect 8.2 million people, with the cost of care reaching up to 100 billion dollars, showing the significance of wound care.^1^ While suturing and stapling are considered gold standards for treatment, they may not always provide an effective solution. Large wounds, for instance, cannot always be completely covered by sutures and stapling, leading to the leakage of body fluids and air.^3^ Additionally, suturing requires advanced skills and surgery time, and it can lead to infections and complications.^2,4^ This is especially critical for emergency cases where rapid intervention is crucial, making suturing less favorable.^5^ While stapling is an easy and simple process, it demands excessive mechanical forces to secure the staples, and they need to be removed after healing.^6^ Furthermore, both suturing and stapling are not suitable for areas with limited access to the wounded area.^2^

Tissue adhesives, particularly biocompatible adhesive hydrogels, are promising alternatives to suturing and stapling. They can seal the wound without leakage and promote wound healing and tissue regeneration.^2,7^ Hydrogels are 3D hydrophilic polymer networks, imbibing large amount of water.^8,9^ They are soft and flexible and mimic the mechanical properties of tissues due to their viscoelastic nature. Moreover, hydrogels may present adhesive properties, making them easy to apply on the wounded areas compared to traditional methods.^10^ However, their durability in wet environments is currently limited. Excessive swelling, for instance, causes them to lose their mechanical and adhesive properties over time. Moreover, such swelling may result in tissue compression and patient discomfort.^11^ Degradation is an additional concern, when hydrogels are used over an extended time in the body, as it can generate a toxic byproduct.^2^ Therefore, addressing the durability of the adhesive hydrogels is critical to ensuring their effectiveness as a treatment option.

Recently, we proposed a series of adhesive hydrogels based upon hydroxyethyl acrylamide (HEAam) and showed their potential use to correct tracheomalacia in a proof-of-concept study.^12^ These hydrogels exhibited excellent adhesive properties on the rabbit trachea surface. However, these hydrogels lack durability under wet conditions, a common problem in adhesive hydrogels, which results in deterioration in both adhesive strength and mechanical characteristics over time. In this work, we introduced PEG-NHS (max. 2 wt %) into the previously developed hydrogel network to enhance their durability in wet environments. PEG-NHS incorporation increased the adhesion strength to up to 50% by forming covalent bonds with the tissue surfaces along with multiple physical interactions. Furthermore, this addition enhanced the mechanical properties, reduced swelling, and increased the degradation time of the hydrogels under enzymatic and oxidative conditions. Importantly, the addition of PEG-NHS did not exhibit any toxicity. To evaluate its clinical potential, we performed *ex vivo* experiments to correct tracheomalacia, a clinical condition characterized by an inadequate mechanical support of the cartilage rings and excessive collapse of trachea during breathing. The addition of PEG-NHS significantly reduced the risk of collapsing by providing stronger mechanical support to the trachea compared to the absence of PEG-NHS. These findings demonstrate that PEG-NHS incorporation can address durability concerns in adhesive hydrogels and offer new possibilities in biomedical applications.

## Results and Discussion

**Figure 1:**
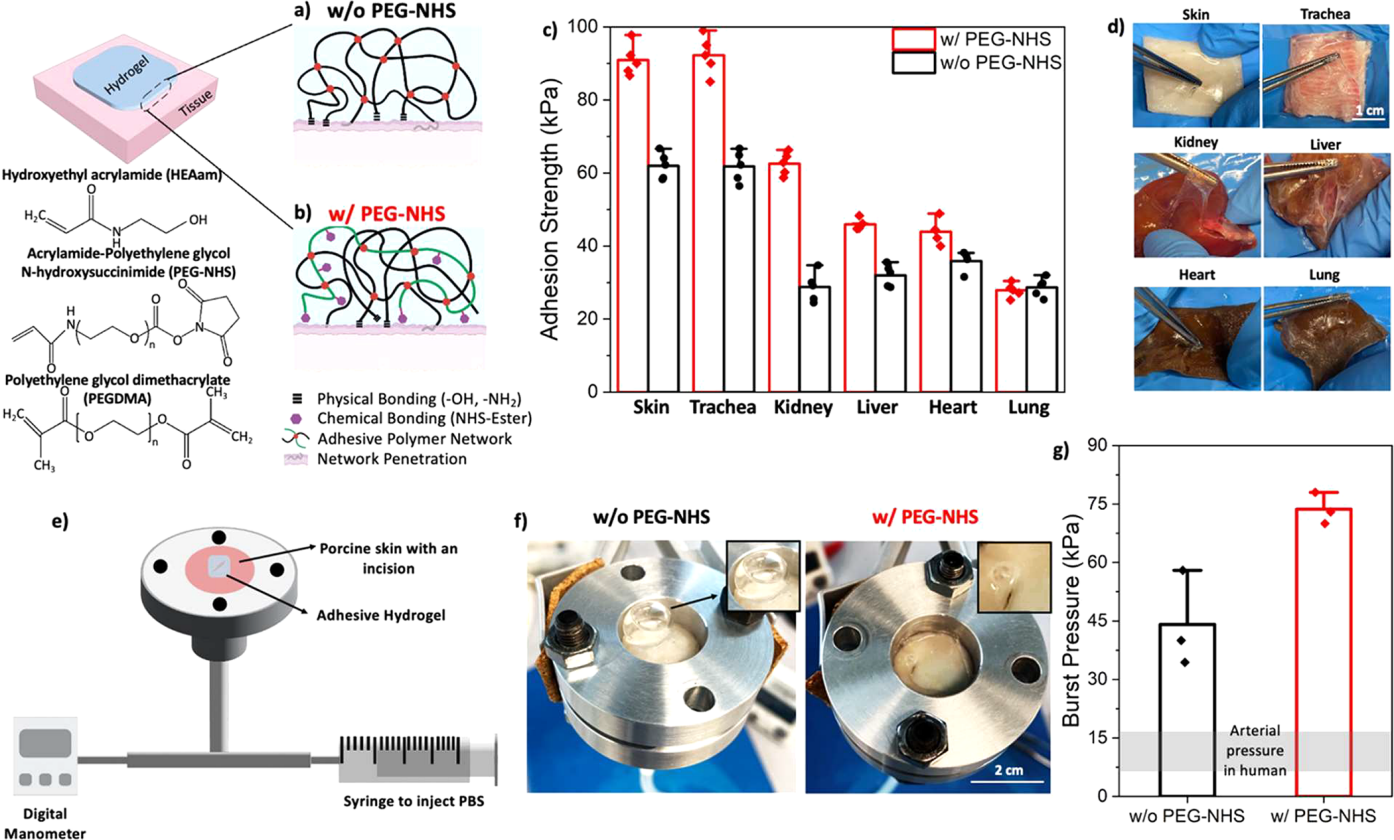
Schematic for (a) w/o PEG-NHS hydrogels where adhesion of the hydrogels results mainly from physical interactions and bulk network penetration, (b) w/ PEG-NHS hydrogels where adhesion performance enhanced is due to synergistic effect of chemical, physical interactions and bulk network penetration. (c) Shear adhesion strength of w/ and w/o PEG-NHS hydrogels on several biological tissues. (d) Representative adhesion pictures of w/ and without PEG-NHS on several tissues. (e) Schematic of the custom-made burst pressure setup. (f) Pictures of the hydrogels forming bubbles due to bursting. (g) Burst pressure strength of w/ and w/o PEG-NHS hydrogels on porcine skin compared to typical arterial pressure in human. Data are represented as the mean ± SEM.

Adhesive hydrogels were synthesized using hydroxyethyl acrylamide (HEAam) as the main polymer network and poly(ethylene glycol) dimethacrylate (PEGDMA) as the cross-linker. The photoinitiator, lithium phenyl-2,4,6-trimethylbenzoylphosphinate (LAP), was used to achieve covalent cross-linking and create a 3D polymeric network of a hydrogel, as depicted in Figure 1a. To determine the optimal monomer and cross-linking concentration for superior adhesion performance, gelatin-coated glass slides were used to screen different formulations. The highest shear adhesion strength and optimal bulk properties were achieved with a blend of 40 wt % HEAam and 2 wt % PEGDMA, following a two-step polymerization approach.

The adhesive properties of the hydrogels depends on the functional groups presents in the polymer networks as well as their bulk mechanical characteristics.^13,14^ These properties of hydrogels dictate the surface interaction with substrates, including gelatin-coated glass slides and tissues. Hydrogels that possess a higher number of functional groups exhibit stronger interactions with the substrate (Figure S1a.). However, once the optimal polymer concentration is reached, further improvements do not occur due to saturation of the interactions between two surfaces (Figure S1a.). This explains why hydrogels with 40 wt % HEAam showed higher adhesion performance than the hydrogels with lower monomer concentration on the glass surface.^12^ On the other hand, covalent cross-linking affects bulk mechanical properties, but excessive cross-linking makes the hydrogel brittle, reducing the adhesion. Therefore, increasing the concentration of PEGDMA above 2 wt % did not yield further improvement in the adhesion strength of the hydrogels (Figure S1b). HEAam-based hydrogels have the ability to form multiple physical interactions and network penetration with corresponding surfaces thanks to the presence of −OH, and −CONH groups in the polymer network. Although these multiple physical interactions can be as strong as a few chemical interactions, they are unstable and reversible in nature, which may not be advantageous for certain long-term biomedical applications.

To address the stability issue and enhance the durability of HEAam-based hydrogels, we chose to incorporate acrylamide poly(ethylene glycol) *N*-hydroxysuccinimide (PEG-NHS) moieties. These moieties can form permanent covalent bonds with tissue surfaces,^15^ in addition to the multiple physical interactions, as illustrated in Figure 1b. Specifically, *N*-hydroxysuccinimide (NHS) is an activated ester that has a propensity to form amide bonds with highly nucleophilic primary amine groups.^16^ These primary amine groups are abundantly present on biological tissue surfaces, typically originating from lysine residues.^2^ This has been shown in a recent study published by He et al. in which the authors developed an injectable adhesive hydrogel containing acrylic acid-NHS (AA-NHS) and observed a 2- to 3-fold of increase in adhesive properties.^17^ Additionally, tissue surfaces also contain carboxylic acids (from glutamic acid), thiols (from cysteine), and imidazole (from histidine) which can engage in multiple physical interactions with the −OH and −CONH- groups of the HEAam.^2,18^ Based on the data obtained from gelatin-coated glass slides, we found that a maximum of 2 wt % PEG-NHS could be incorporated into the HEAam-based hydrogels. Higher amounts of PEG-NHS resulted in increased brittleness of the hydrogels (Figure S1c). As a result, two hydrogel formulations were selected for further studies and comparison: one, referred to as ***w/o PEG-NHS*** hydrogel (where *w/o* stands for “*without*”) consisting only of HEAam (40 wt %) and PEGDMA (2 wt %), and the other hydrogel, referred to as ***w/ PEG-NHS*** hydrogel (where *w/* stands for “*with*”) consisting of HEAam (38 wt %), PEGDMA (2 wt %), and PEG-NHS (2 wt %).

To evaluate the effect of PEG-NHS on adhesion, we conducted shear adhesion tests of w/ and w/o PEG-NHS hydrogels on various tissue surfaces, including porcine skin and rabbit trachea as well as bovine kidney, liver, heart, and lung (Figure 1c,d). The results showed that w/o PEG-NHS hydrogel achieved an adhesion strength of ∼60 kPa on the skin and trachea surfaces. In contrast, w/ PEG-NHS hydrogel showed a significantly higher adhesion strength of ∼90 kPa on these surfaces. Similarly, w/ PEG-NHS hydrogel showed superior adhesion strength on kidney (∼60 kPa), liver (∼45 kPa), heart (∼45 kPa), and lung (∼25 kPa) compared to the case w/o PEG-NHS (∼25–30 kPa).

It is important to note that the adhesion strength of hydrogels can be very dependent on the type of tissue and the presence of body fluids. As mentioned above, this stems from the quantity of the functional groups present on the tissue surfaces as well as mechanical properties of the tissues.^13^ The liver, lung, kidney, and heart are covered by a serous membrane, which is composed of cells secreting serous fluids and lubricating the surface. The presence of this surface lubrication can affect the adhesion strength of the hydrogel.^19,20^ That is why we observed lower adhesion values on these tissues with both hydrogel formulations. The adhesion performance of the developed hydrogels was also compared to a commercially available tissue adhesive called TISSEEL(Baxter), specifically on rabbit trachea surface.^12^ The results showed that w/o PEG-NHS hydrogel and w/ PEG-NHS hydrogel exhibited six-times and nine-times higher shear adhesion strength than TISSEEL, respectively.

We further conducted a burst pressure test to evaluate the endurance of the hydrogels against the pressure exerted by body fluids. As an example, hydrogel adhesives developed to seal arteries have to withstand ∼200 mmHg before rupturing, while the burst pressure threshold of ∼67 mmHg is required for corneal incisions.^21^ For this purpose, we utilized a custom-made burst pressure setup,^13^ as shown in Figure 1e. Briefly, an incision was made on porcine skin, and it was securely fixed to the setup. The precursor of the hydrogels was poured onto the incision area, and the preformed hydrogel was placed on top. A second polymerization step was performed for 5 min to adhere the hydrogel on the porcine skin. Subsequently, PBS was injected through a syringe that is connected to the burst pressure setup. The pressure exerted on the hydrogel was recorded by using a digital manometer. As shown in Figure 1f,g, w/o PEG-NHS hydrogel exhibited a burst pressure strength of ∼45 kPa and formed a large bubble before bursting, as observed in Figure 1g. Interestingly, w/ PEG-NHS hydrogel performed ∼1.5 times better compared to w/o PEG-NHS hydrogel. It sustained a burst pressure of ∼75 kPa and formed only a small bubble, indicating an improved resistance to bursting. It should be noted that burst pressure values of both hydrogels are much higher than the typical biological pressures; i.e., the typical arterial pressure in humans is around 10–16 kPa.^21^ These results suggest that w/ and w/o PEG-NHS hydrogels hold great potential for tissue adhesive applications.

**Figure 2:**
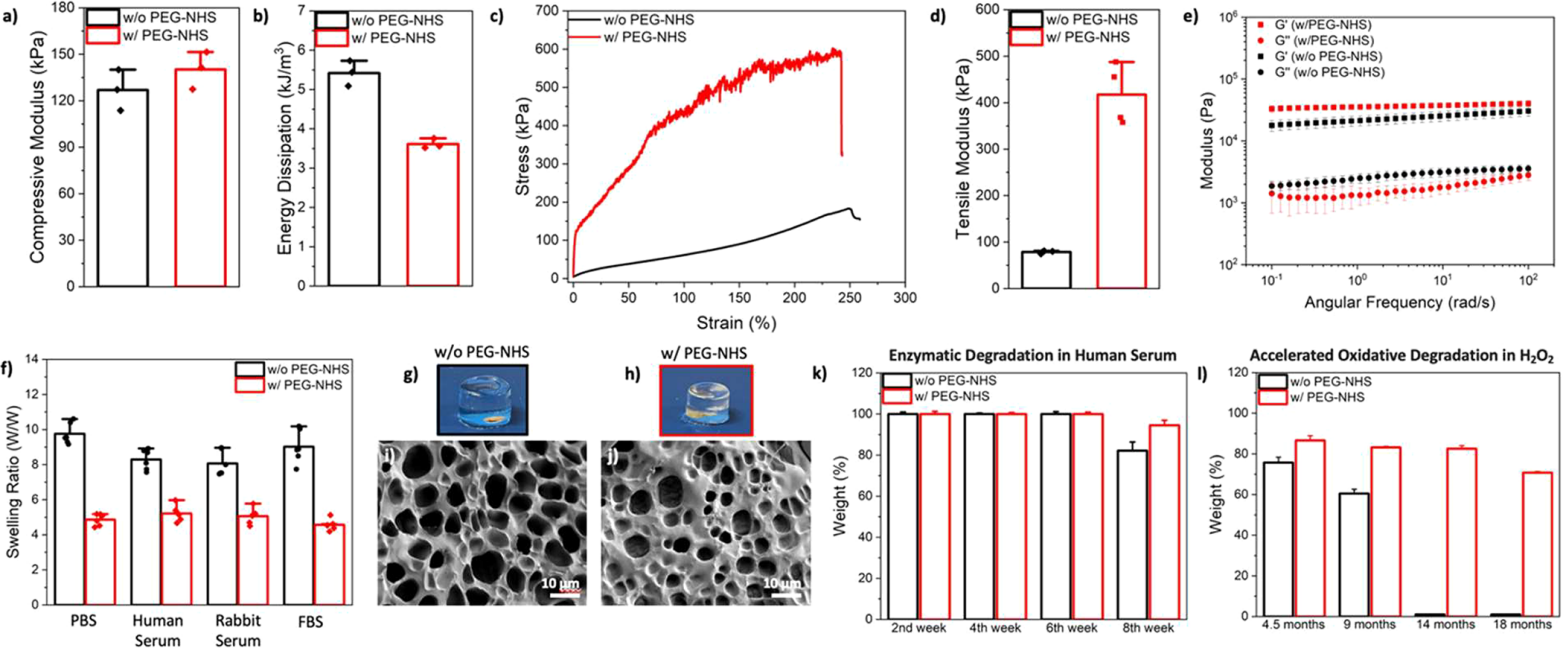
(a) Compressive modulus of the hydrogels (*n* = 3), (b) Energy dissipation of the hydrogels (*n* = 3), (c) Stress–strain curves of the hydrogels under tension (*n* = 5), (d) Tensile modulus of the hydrogels (*n* = 5), (e) Oscillatory rheological data: frequency sweep to analyze the shear modulus of the hydrogels (*n* = 3), (f) Swelling degree of the hydrogels in different media (*n* = 3), (g, h) Representative pictures of swollen hydrogels in PBS. (i, j) SEM micrographs of the freeze-dried hydrogels; scale bar is 10 μm. (k) Enzymatic degradation of the hydrogels in simulated human serum up to 8 weeks (*n* = 3). (l) Accelerated degradation of the hydrogels in aqueous H_2_O_2_ at 80 °C for up to 4 weeks, which corresponds to 18 months (*n* = 3). Data are represented as mean ± SEM.

We then examined the mechanical properties of the hydrogels. As shown in Figure 2a, the compressive modulus of w/o PEG-NHS hydrogel is ∼120 kPa, while that of w/ PEG-NHS hydrogel is ∼150 kPa. We also determined the hydrogel’s dissipative properties (Figure 2b). We observed that w/o PEG-NHS hydrogel (∼5.5 kJ/m^3^) dissipated ∼1.5 times more energy than w/ PEG-NHS hydrogel (∼3.5 kJ/m^3^). This phenomenon may be attributed to the presence of PEG-NHS moieties, which are expected to enhance both physical and chemical interactions within the bulk HEAam-polymer network of the hydrogel. PEG-NHS incorporates unsaturated acrylamide units as well as NHS units within a single molecule. During polymerization, acrylamide is copolymerized with HEAams, leaving the polymer network with NHS backbones and functionalities present on HEAam.^18^ The presence of NHS units is anticipated to further enhance physical interactions with functionalities (−OH, amide, etc.) on the HEAam–polymer network.^22^ Consequently, this should lead to an increase in the number of physical and chemical cross-linking points, resulting in a stiffer and more rigid network structure of w/ PEG-NHS hydrogel.^18,23^ This stiffness can also explain the comparably lower dissipative property of w/ PEG-NHS hydrogel than w/o PEG-NHS hydrogel.

As a next step, the tensile stress–strain behavior of the hydrogels was analyzed, and the results are given in Figure 2c,d. Hydrogel w/ PEG-NHS showed a significantly higher elastic modulus, up to five times greater, compared to w/o PEG-NHS hydrogel. Such difference in tensile modulus is attributed to the stiffer nature of w/ PEG-NHS hydrogel, which has higher chemical and physical intramolecular cross-linking points due to the addition of PEG-NHS as mentioned before. However, the variation in compressive and tensile modulus, known as tensile-compression asymmetry, is commonly observed in soft polymeric materials.^24^ This is more pronounced in w/ PEG-NHS hydrogel, which can be related to its higher cross-linking density. Under compressive load, the polymer chains can buckle and cross-linking points may weaken, which leads to localized softening.^25^ This means that polymeric chains are dangled, and the stresses in each chain reduce significantly. On the other hand, the chains and cross-linking points play a more active role under tensile load, contributing to the overall deformation behavior. Therefore, the more cross-linking points the hydrogel has, the more buckled or active cross-linking it has under compressive or tensile load.^24,25^ This could lead to a drastic difference between the tensile and compression moduli in a highly cross-linked network of a hydrogel. The stress–strain curve of w/ PEG-NHS hydrogel shows irregularities (plastic discontinuities) after 75% strain, and completely breaks at approximately 250% strain (Figure 2c). These irregularities stem from localized fractures or slips in the structure, commonly seen in brittle materials.^26^ However, despite being more brittle, w/ PEG-NHS hydrogel may still offer sufficient flexibility and elasticity as tissue adhesive for various tissues in the human body. For example, it was determined that the ultimate tensile strain of human skin is ∼55%, whereas human liver stretches only up to 35%.^27^

Similar to the tensile test, the oscillatory rheological study confirmed that w/ PEG-NHS hydrogel is stiffer than w/o PEG-NHS hydrogel. The frequency sweep, conducted from 0.1 to 100 rad/s angular frequency and at 0.1% strain, revealed that the storage moduli (*G*′) and the loss moduli (*G*″) of w/ PEG-NHS hydrogel is higher than those of w/o PEG-NHS hydrogel, as depicted in Figure 2e.

Hydrogels are 3D polymeric networks with high hydrophilicity, enabling them to absorb substantial amounts of water, which can be advantageous depending on the applications. Nevertheless, excessive swelling can be detrimental when hydrogels are used as tissue adhesives. As the hydrogels swell, they tend to interact more with water molecules rather than the functional groups present on the tissue surface, resulting in a reduction of their intrinsic adhesion properties.^28^ Therefore, we sought to investigate the swelling ratio of the hydrogels in different media. Phosphate buffered saline (PBS), human serum, rabbit serum, and fetal bovine serum (FBS) were chosen for the experiments. We found that the swelling degree of the HEAam hydrogels reduced by half upon adding PEG-NHS (2 wt %), regardless of the swelling media (Figure 2f). That is because the swelling degree of hydrogels is determined by the mesh size and cross-linking density. With the presence of acrylamide and NHS moieties, w/ PEG-NHS hydrogel has smaller mesh sizes and therefore a lower swelling degree. To provide further explanation, scanning electron microscopy (SEM) was used to examine the structures of both hydrogels. Figure 2i,j show that w/o PEG-NHS hydrogel has overall bigger pore size compared to w/ PEG-NHS hydrogel and has therefore less compact structure. This structural difference should account for the reduced swelling degree observed in w/ PEG-NHS hydrogel.

We further examined the degradation behavior of the hydrogels under oxidative and enzymatic conditions to determine their durability inside the body, which is critical for long-term applications. Hydrogels primarily experience the hydrolytic and oxidative degradation within body.^29^ Hydrolytic degradation can be enzymatic or nonenzymatic, involving water and enzymes in the process.^30,31^ Hydrolytic degradation test was conducted on w/ and w/o PEG-NHS hydrogels in various environment for up to two months at 37 °C, including PBS, human serum, rabbit serum, and FBS. The hydrogels showed mass loss only in simulated human serum, which contains Lipases and Lysozymes (Figure 2k). After 60 days of experiment, w/ and w/o PEG-NHS hydrogels showed 5.5% and 17.8% mass loss in human serum, respectively. This is because lipase catalyzes the hydrolysis of ester bonds present in both hydrogel’s networks due to PEGDMA and PEG-NHS.^32^ Thus, both hydrogels experienced a mass loss due to ester bond degradation. However, w/ PEG-NHS hydrogel degraded approximately three times less compared to w/o PEG-NHS hydrogel. The higher cross-linking density of w/ PEG-NHS hydrogel should be the reason for its lower mass degradation. The increased cross-linking should provide more stability and resistance to degradation, making the hydrogel less susceptible to bond cleavage by water and enzymes.

Macrophages, which are responsible for immune reaction of the body, can produce reactive oxygen species in response to the degree of inflammation when foreign materials are implanted in the body.^29^ These highly reactive oxygen species (ROS) can cause oxidative degradation of the hydrogels. Thus, we also investigated the oxidative degradation behavior of w/ and w/o PEG-NHS hydrogels in H_2_O_2_ solution. To observe the long-term degradation profile, we conducted experiments for 4 weeks at 80 °C, which accelerates the kinetics of the degradation reaction.^33^ According to the ASTM-WK4863 standard,^33^ there is a correlation between temperature increase and the degradation behavior as shown below:
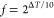
where Δ*T* = *T* – *T*_ref_. *T* is the temperature at which the degradation is conducted, and *T*_ref_ is the reference temperature (in our case, 37 °C, the body temperature).

Therefore, according to the formula, accelerated degradation at 80 °C should increase overall degradation time by a factor of 20. A four week experiment corresponds to 1.5 years, providing a reasonable time scale for studying the long-term hydrogel degradation. It should be noted that accelerated degradation was conducted only in H_2_O_2_ due to the denaturation of the enzymes at temperatures above 50–55 °C.^34^Figure 2l illustrates that w/o PEG-NHS hydrogel degraded completely between 9- and 14-months period, whereas w/ PEG-NHS hydrogel showed only around 25% degradation in the entire 18 months. This result confirms that 2 wt % PEG-NHS addition to the HEAam hydrogel network decreased the degradation rate.

**Figure 3:**
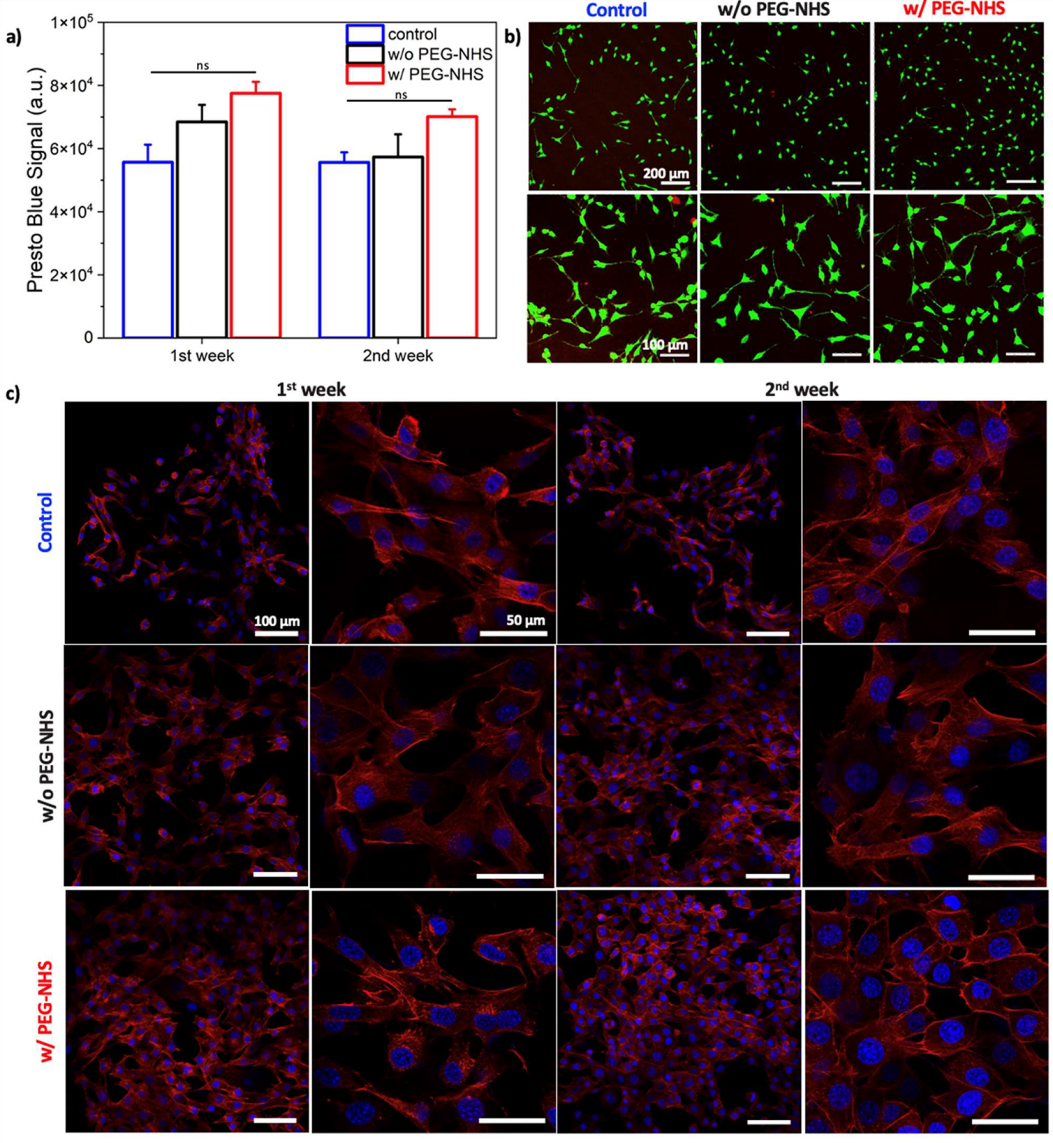
(a) Presto Blue signal of the mouse fibroblast cells incubated with regular cell culture medium (control) and conditioned medium; where w/ and w/o PEG-NHS hydrogels incubated for 2 weeks, (*n* = 5). (b) Live (green) and dead (red) cells incubated with the control or conditioned medium of hydrogels. Pictures represent the data of 2 weeks of incubation, (*n* = 3). (c) Fluorescent staining of cells with DAPI (nuclei, blue) and F-actin (cytoskeleton, red) incubated with normal (control) and conditioned cell culture medium (hydrogels) for 2 weeks, (*n* = 3). Data are represented as mean ± SEM.

We then examined the cytotoxicity of hydrogels by assessing the proliferation and viability of embryonic mouse fibroblast cells. To this end, the hydrogels were incubated for 2 weeks in a cell culture medium. At the end of each week, the medium where the hydrogels were incubated was collected and referred to as conditioned medium. Fibroblast cells were then exposed to this conditioned medium to check the toxicity of the hydrogels (see Experimental Methods for the detailed procedure). Fibroblasts were subsequently stained with Presto Blue, a cell viability indicator employing the reducing capabilities of viable cells to quantitatively assess cell proliferation. Figure 3a shows that the cells incubated with the conditioned media had similar Presto Blue signals compared to the cells incubated with regular cell culture medium (control), showing no statistical difference between the experimental groups after 1 and 2 weeks of incubation. Furthermore, a Live/Dead assay confirmed that cell populations exposed to w/ and w/o PEG-NHS hydrogels showed a similar number of live cells (green) as the control group (Figure 3b). Moreover, we did not observe dead cells (red) in the hydrogel groups.

To assess the cell morphology, we stained the cells with DAPI (for cell nuclei, in blue) and F-actin (for cell cytoskeleton, in red). As depicted in Figure 3c, mouse fibroblast cells adhered and spread well when incubated with the conditioned medium of the hydrogels. Actin filaments of the cells were visible for all of the experimental groups, which is vital for cell shape and movement. Overall, we did not see any cytotoxic effect and confirmed normal cell morphology in the presence of the conditioned medium, indicating that w/ and w/o PEG-NHS hydrogels are biocompatible and can be useful for tissue adhesive applications.

**Figure 4:**
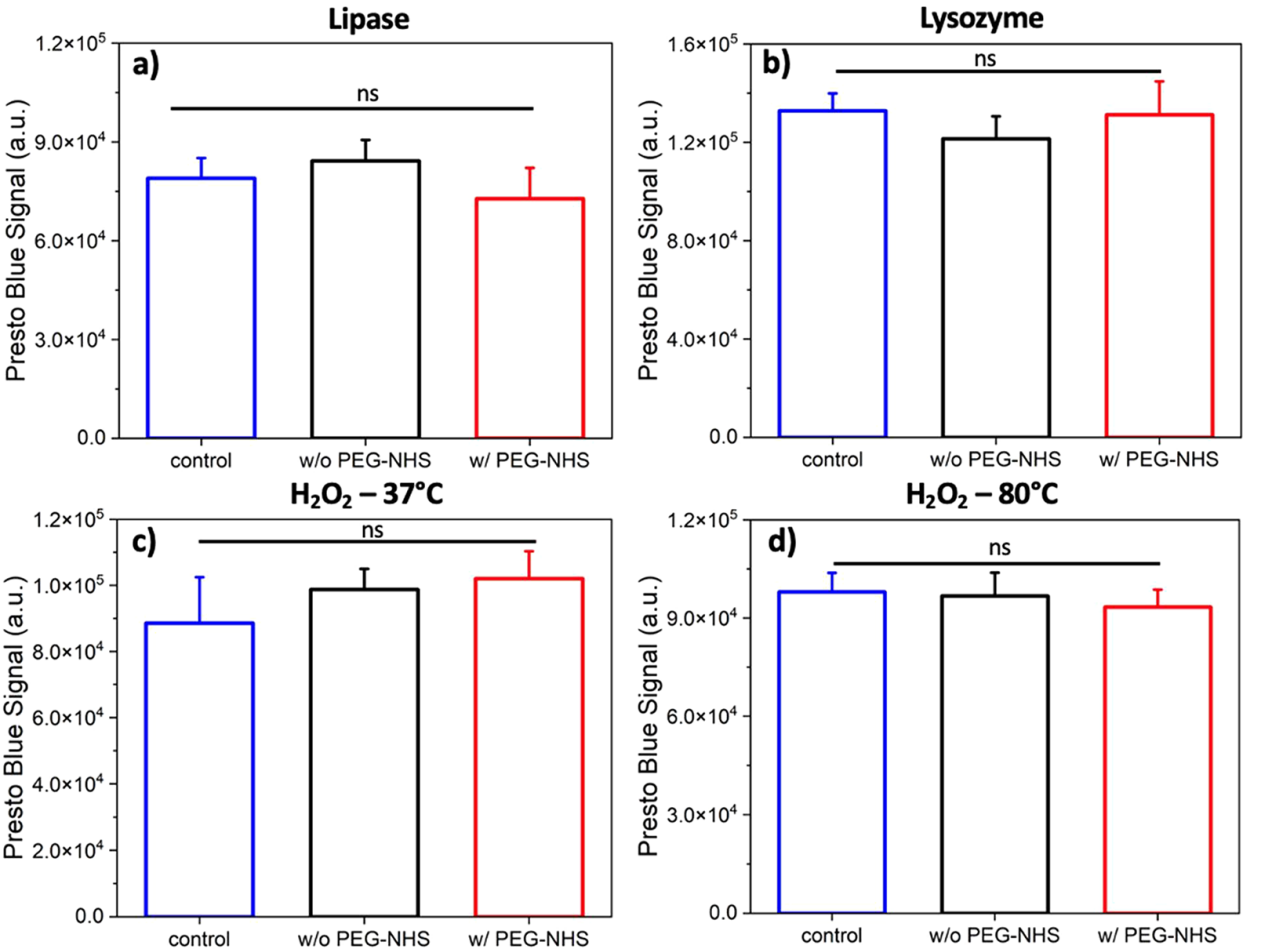
Presto blue signal of the fibroblast cells incubated with diluted released-products of w/ and w/o PEG-NHS hydrogels after their degradation in (a) lipase, (b) lysozyme, and H_2_O_2_ at (c) 37 °C and (d) 80 °C compared to the control group. Data are represented as mean ± SEM (*n* = 5).

During the degradation process, hydrogels tend to release low molecular weight polymeric chains, oligomers, and small molecules to the surroundings.^29^ Such released products could be toxic to the cells. Thus, we also sought to examine the toxicity of the degradation products of w/ and w/o PEG-NHS hydrogels. To this end, fibroblast cells were incubated with released products which were diluted in cell culture medium (1:9, see Experimental Methods for the detailed procedure). The results in Figure 4 show no statistical difference between the Presto blue signal of the fibroblast cells incubated with regular culture medium (control) and the diluted released-products of hydrogels, indicating the nontoxicity of the released products of w/ and w/o PEG-NHS hydrogels to fibroblast cells.

**Figure 5:**
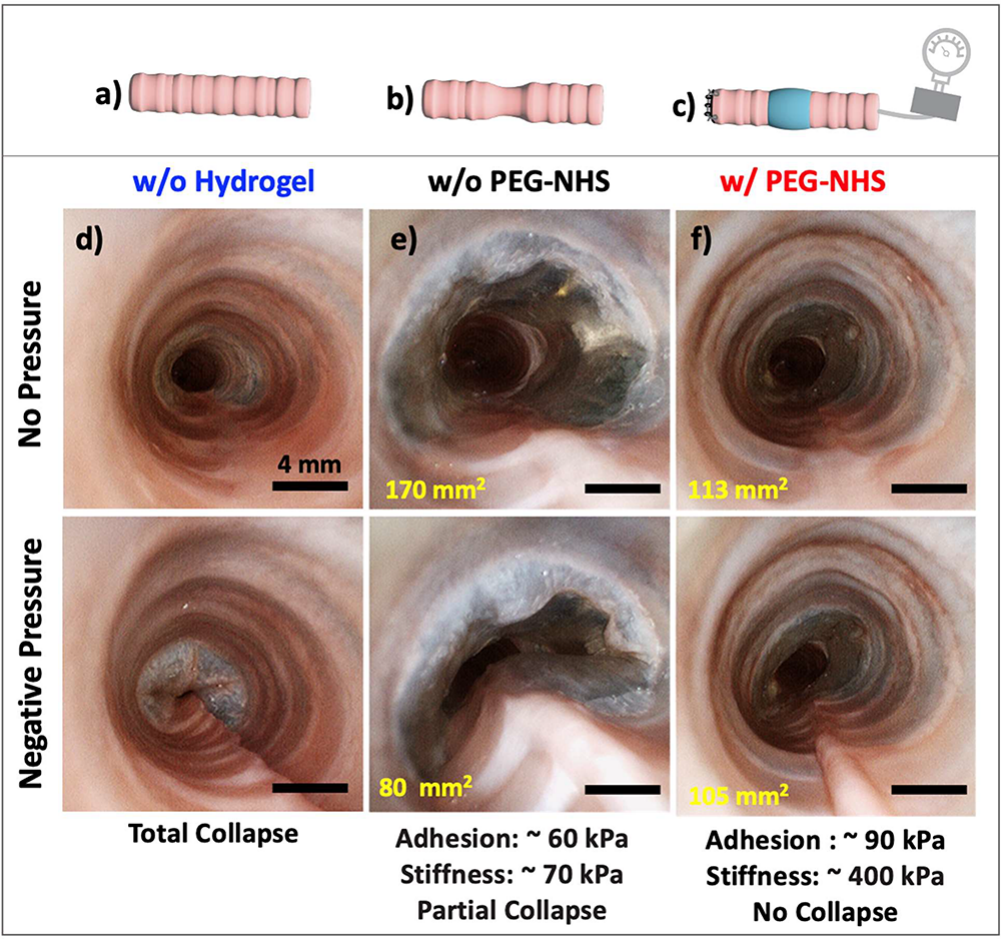
Schematic of (a) the healthy trachea, (b) the malacic trachea after removal of several cartilage rings, and (c) the suction machine attached to the flexible bronchoscope with the hydrogel wrapped around the malacic trachea. (d) Total collapse of the malacic trachea under the applied negative pressure (∼ −5 kPa) without a hydrogel patch. (e) Partial collapse of the malacic airway under the applied negative pressure of (∼ −5 kPa) when w/o PEG-NHS hydrogel patch is wrapped around the malacic trachea. (f) No collapse was observed when w/ PEG-NHS hydrogel patch is placed extraluminally around the trachea. Please note that the values in bronchoscope images represent the open airway area before and after negative pressure.

Tracheomalacia is a clinical condition where the airway collapses due to weakness in the tracheal cartilage and muscles, posing a life-threatening risk for newborns.^35^ Current treatments involve surgery and stenting.^36^ However, surgery may not be suitable for severe cases, and endoluminal stents can cause airway blockage and inflammation.^37^ Additionally, stent materials are too rigid to accommodate the trachea’s natural movement during breathing, hindering the tissue growth.^38^ To address these challenges, we proposed a new approach in our recent study: using an adhesive hydrogel patch to support the weakened trachea and prevent collapse.^12^ To this end, we performed *ex vivo* studies with rabbit tracheas (consisting of a 10 cm long trachea and the larynx) to examine the potential use of the HEAam-based adhesive hydrogels for tracheomalacia treatment. Significant portions of 4–5 cartilage rings of the healthy rabbit trachea (Figure 5a) were carefully removed maintaining the endotracheal mucosa intact and avoiding any tears in it to create tracheal fragility and malacia (Figure 5b). The distal trachea was closed with a suture, and a 3.8 mm flexible bronchoscope was passed through the larynx. Negative pressure measured on a manometer was applied via the bronchoscope, and the proximal laryngeal end was maximally closed by applying digital pressure to create a maximal water-tight effect on the malacic trachea with and without wrapping a hydrogel patch around it (Figure 5c). We observed that a malacic trachea without a hydrogel patch collapsed completely under applied negative pressure due to the lack of mechanical support from the tracheal cartilage rings, as shown in Figure 5d. When the hydrogel patch w/o PEG-NHS is wrapped around a malacic trachea, the collapse was reduced by 50%, see Figure 5e. Remarkably, we achieved a significant improvement in correcting a malacic trachea by using the hydrogel patch with PEG-NHS. This hydrogel patch provided firm support to the trachea, resulting in no collapse under the applied negative pressure, leading to approximately 90% improvement in the open area of the airway (Figure 5f). This indicates that hydrogels w/ and w/o PEG-NHS have potential to correct malacic trachea. Hydrogel w/ PEG-NHS can be more effective for the severe malacic cases where collapse may go higher than 75% of airway diameter.^36,39^ This holds great promise, and we eagerly anticipate developing a technique to observe this effect in an animal model with severe malacia characterized by a high degree of lumen collapse for our ongoing studies.

## Conclusion

Adhesive hydrogels show promise as alternatives to suturing and staples for complex surgeries and tissue bonding. However, their durability in the presence of body fluids remains an issue. This study presents a simple but effective approach to enhance the adhesion and durability of hydroxyethylacrylamide- (HEAam-) based hydrogels by incorporating PEG-NHS into their structure. We found that hydrogels with PEG-NHS exhibited significantly higher adhesion, almost twice as much as the hydrogels without PEG-NHS on various tissues. Furthermore, the addition of PEG-NHS enhanced the mechanical properties and durability of the hydrogels by reducing swelling and degradation rates in different media. Notably, with and without PEG-NHS hydrogels were found to be nontoxic to fibroblast cells, even when exposed to enzymatic and oxidative conditions. To assess its potential for clinical use, we conducted *ex vivo* experiments to correct tracheomalacia—a condition characterized by insufficient mechanical support of the cartilage rings and excessive collapse during breathing. By incorporating PEG-NHS in the developed hydrogels, we observed enhanced support to the trachea, resulting in a significant reduction in the risk of collapsing compared to hydrogels without PEG-NHS. These results underscore the ability of PEG-NHS to address durability issues in adhesive hydrogels and open up new possibilities for biomedical applications.

## Experimental Methods

### Materials

The chemicals used in this study were obtained from the following sources: *N*-(2-Hydroxyethyl)acrylamide (HEAam) from Chemie Brunschwig AG, lithium phenyl 2,4,6-trimethylbenzoyl phosphinate (LAP) from Sigma-Aldrich, and polyethylene glycoldimethacrylate (PEGDMA, Mn = 20 kDa) from Polysciences. Additionally, acrylamide (Aam)-polyethylene glycol (PEG, 1 kDa)-NHS was acquired from Abbexa. Young male rabbit tracheas (5–6 kg) were provided by Delimpex AG, Pfäffikon, Switzerland. Moreover, porcine skin, bovine heart, kidney, lung, and liver samples were obtained from a local slaughterhouse.

### Fabrication of HEAam-Based Adhesives

HEAam-based hydrogel without PEG-NHS was developed using a method published earlier by our group.^12^ In brief, HEAam (20–50% w/v), PEGDMA (1 to 3% w/v), and photoinitiator (PI) LAP (0.05% w/v) were dissolved in PBS using vortex in the absence of light. The precursor solution was poured into a 15 × 15 × 0.7 mm^3^ custom-made Teflon molds and covered with plastic slides. To initiate the photopolymerization process, the molds were exposed to 405 nm light at an intensity of 3 mW cm^–2^ for 5 min, resulting in the formation of an adhesive hydrogel patch. Similarly, HEAam with PEG-NHS was obtained by mixing 38% (w/v) HEAam, 2% (w/v) PEG-NHS, 2% (w/v) PEGDMA, and PI (0.05% w/v).

### Adhesion Measurements

The shear adhesion of the adhesive hydrogels on their respective surfaces was evaluated following the guidelines outlined in the ASTM F2255 standards.^40^

#### (a) Glass Adhesion

The preformed hydrogel patch of 15 × 15 × 0.7 mm^3^ was obtained according to the described method in the fabrication section (Figure S2, step 1–2). 100 μL of same precursor solution was poured on the preformed hydrogel, which was already adhered to a gelatin-coated glass slide (25 × 75 × 1 mm^3^) (Figure S2, step 3). Another gelatin-coated glass slide was then placed on the hydrogel surface followed by the second photopolymerization (Figure S1, step 4). After the second polymerization, adhesion measurements were performed using 50 N load cell connected to Instron E3000 mechanical testing machine with a constant loading rate of 1 mm/s. Shear adhesion strength of the hydrogels was calculated dividing the maximum load by the surface area of the hydrogel patch.

#### (b) Soft Tissue Adhesion

Before adhesion measurement, all tissues were hydrated in PBS for 45 min and then cut to the dimensions of 10 × 25 mm^2^. Porcine skin and rabbit trachea were attached to a glass slide using *Superglue* (Loctite 401). To create the preformed hydrogel, a precursor solution was poured onto a Teflon mold (15 × 15 × 0.7 mm^3^) and covered with a plastic slide. After 5 min of polymerization, the preformed hydrogel was carefully detached from the plastic slide. Then, 100 μL of the precursor solution was applied to the wet trachea or skin surfaces, and the preformed hydrogel was placed gently on top without introducing any air bubbles. Another 100 μL of the precursor solution was added to the upper surface of the hydrogel and covered with a glass slide. A second polymerization step was then performed. The shear adhesion strength of the adhesive hydrogels was measured by using an Instron E3000 with a 50 N load cell, applying a constant loading rate of 1 mm/s. The calculation of the shear adhesion strength followed the method described for glass adhesion.

For the heart, liver, lung, and kidney, the adhesion measurement was conducted differently due to the slippery surface of these tissues. To this end, a free-standing adhesion method was used, as depicted in Figure S3. Initially, two preformed hydrogels were prepared and attached to a glass slide, following the same procedure as that described earlier. Then, 100 μL of the precursor solution was applied to the wet tissue surfaces. The preformed hydrogel on the glass slide was carefully placed onto the tissue surface (see Figure S3a). Subsequently, the second polymerization step was performed (Figure S3b). This process was repeated on the other side of the wet tissue (Figure S3c,d). Once the polymerization was complete, two preformed hydrogels and the free-standing tissue were secured to the adhesion setup, and the measurements were carried out using the same method as described previously (see Figure S3e).

### Burst Pressure

Porcine skin was thawed and hydrated for 45 min before the burst pressure measurements. A 3 × 3 cm^2^ of skin piece was cut and 0.5 cm of incision was created with a scalpel. Skin piece was mounted to the burst pressure setup, which is shown in Figure 1e. Meanwhile, adhesive hydrogels with and without PEG-NHS having dimensions of 15 × 15 × 0.7 mm^3^ were prepared as mentioned earlier. Subsequently, 100 μL of precursor solution of respective hydrogels were poured onto the skin surface and second polymerization was performed. The device was connected to the syringe which injects the PBS solution, and the digital manometer was connected to read the burst pressure. Peak pressure, without pressure loss, was considered as a burst pressure strength. The test for each hydrogel was repeated three times.

### Compression Test

Disk-shaped samples (o.d. 8.5 mm) of adhesive hydrogels were prepared following the fabrication protocol mentioned above. The compressive modulus and energy dissipation of the hydrogels were measured by using an Instron E3000 linear testing machine (Norwood, MA, USA) equipped with a 250 N load cell. During the testing process, a strain rate of 0.1 mm/s was applied, and the samples were compressed up to 20% strain. The compressive modulus of the hydrogels was calculated by considering the linear region of the stress–strain curve between 5% and 10% strain, using the linear regression method (*n* = 3). The energy dissipation of the hydrogels was determined by the area enclosed by a hysteresis loop in the stress–strain curve of the compressed sample.

### Tensile Test

The tensile tests of dog-bone-shaped hydrogel specimens (1 mm thickness, 2 mm neck width, and 12 mm gauge length) were carried out using ZwickRoell uniaxial testing machine (Ulm, Germany) equipped with a 100 N load cell. The specimens were placed within the grippers and elongated at a loading rate of 0.1 mm. s^–1^ until breaking. The elastic modulus (tensile modulus) of the hydrogels was calculated on the initial linear slope of the tensile stress–strain curves at 10–15% strain (*n* = 5).

### Rheology

The rheological properties of the hydrogel were investigated using Anton Paar MCR102 (Graz, Austria) in parallel plate configuration with a disc diameter of 25 mm. Linear viscoelastic region of the samples was determined at constant angular frequency of 1 rad/s applying shear strain from 0.1% to 100%. Measurements were repeated twice. Then, frequency sweep test was carried out at 0.1% strain by changing the angular frequency from 0.1 to 100 rad/s to obtain storage (*G*′) and loss (*G*″) modulus of the samples (*n* = 3).

### Swelling Measurement

Disk-shaped hydrogel samples (o.d. 4 mm, 3 mm in height) were synthesized using the previously mentioned method. The initial weight of each hydrogel sample was measured, after which they were immersed in different media, namely, PBS (Gibco 10010023), Fetal bovine serum (Gibco, 26140079) Rabbit Serum (Gibco, 16120107) and Human Serum. Human serum was prepared using Amano Lipase (0.005 mg/mL in PBS, Sigma-Aldrich, 534641) and Lysozyme (0.013 mg/mL in PBS, Sigma-Aldrich, 62971). Three replicates were prepared for each solution. Weight swelling ratios of the samples were calculated after 3 days according to
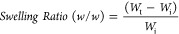
where *W*_t_ is the swollen weight of the hydrogels after 3 days inside the medium and *W*_i_ is the initial weight of the hydrogel.

### Scanning Electron Microscopy (SEM)

Hydrogels were kept at −80 °C for 1 day and placed into a lyophilizer (LABCONCO, Kansas City, MO, USA) for 1 week. Freeze-dried samples were coated by Au–Pd alloy (6 nm) using a Quorom Q150TPlus high-resolution sputter coater (Lewes, United Kingdom) before the analysis. The topology of the hydrogels was investigated by using a scanning electron microscope (GEMINI SEM 300, Carl Zeiss, Germany) equipped with a secondary electron detector. During surface characterization, the accelerating voltage was kept at 3 kV.

### Degradation Study

Disk shaped hydrogel samples (o.d. 4 mm, 3 mm in height) were prepared for degradation experiments. These samples were then placed in a −80 °C freezer for 1 day. The next day, lyophilization (using a LABCONCO lyophilizer, Kansas City, MO, USA) was performed for 1 week. After lyophilization, the dried weight of each sample was measured. The lyophilized samples were then placed inside 12-well plates or 50 mL centrifuge tubes, which were used to seal the samples and prevent evaporation of the degradation medium. In each well or tube, 5 mL of the degradation medium was added. The degradation medium was changed every 3 days to ensure consistent conditions. At the end of the specified degradation time interval, the samples were removed from the degradation medium. They were then washed with PBS, and any excess PBS on the surface was carefully removed using filter paper. Next, the samples were frozen for 1 day and then freeze-dried for 1 week to obtain their dried degraded weight. The following formula was used to calculate degradation:
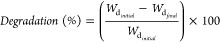
where *W*_d_*initial*__ and *W*_d_*final*__ are the initial and final dried weight of the samples, respectively.

#### (a) Oxidative Degradation

0.003 wt % H_2_O_2_ (ACS reagent, 30 wt % in water, Chemie Brunschwig AG, ACR41188) were used as degradation medium to investigate long-term degradation profile of the hydrogel samples. Samples were placed into 50 mL centrifuge tubes in an 80 °C oven for 1, 2, 3, and 4 weeks. The corresponding degradation time was calculated using following formula:^33^
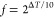
where Δ*T* = *T* – *T*_*ref*_*. T* is the temperature at which the degradation is conducted and *T*_*ref*_ is the reference temperature (in our case, 37 °C, the body temperature). With the 4 week experimental period at 80 °C, degradation was accelerated ∼20 times, corresponding to 600 days.

#### (b) Enzymatic Degradation

Simulated Human Serum (composed of lysozyme (0.013 mg/mL) and lipase (0.005 mg/mL)) was used as degradation medium to observe enzyme driven hydrolytic degradation behavior. The experiment was conducted at 37 °C for 2, 4, 6, and 8 weeks. Samples were taken out every 2 weeks and freeze-dried before measuring the final dried weight. Samples put into PBS were considered as control.

### Biocompatibility of the Hydrogels

#### (a) Metabolic Activities of the Cells

Fibroblast cells derived from mouse embryos (NiH/3T3, ATCC CRL-1658) were used to evaluate the toxicity of the hydrogels. Briefly, the precursor of the adhesive hydrogel was filtered inside the laminar flow and poured into the sterilized disk-shaped molds (o.d. 5 mm). Then, molds were covered with glass slides and polymerization was performed for 5 min inside the laminar flow. After polymerization, the adhesive hydrogels were placed into 24-well plates filled with complete cell culture medium (DMEM supplemented with 10% (v/v) Fetal Bovine Serum, 1% (v/v) penicillin–streptomycin, and 1% (v/v) l-glutamine). Subsequently, the cell culture plate was placed into the incubator (37 °C and 5% CO_2_) for 1 and 2 weeks. After fixed time intervals, hydrogels were removed from the plate. The conditioned medium, where hydrogels were incubated, was put into 96 well-plates containing 1000 cells/well and incubated for 1 day. Next, medium was aspirated from the well-plate, and 100 μL of 10% (v/v) PrestoBlue (A13261, Life Technologies) was put into each well and the plate was incubated for 30 min. After that, fluorescence was measured at 595 nm by using a microplate reader (Wallac 1420 Victor2, PerkinElmer). Toxicity experiments were performed in triplicate using five replicates for each experiment. DMEM solution with 1000 cells/well was taken as control.

#### (b) Live–Dead Assay

Sterile hydrogel samples were prepared as mentioned above and incubated in the complete cell culture medium (DMEM supplemented with 10% (v/v) Fetal Bovine Serum, 1% (v/v) penicillin–streptomycin, and 1% (v/v) l-glutamine) for 1 and 2 weeks under standard cell culture conditions (37 °C and 5% CO_2_). At the end of weeks one and two, conditioned medium was collected. Fibroblast cells were seeded in 12-well plate at a density of 5000 cells/well on the cylindrical sterile coverslips. Then, conditioned medium was added to each well, and the plate was placed into the incubator for 1 day. After 1 day of incubation, medium was aspirated gently from each well. Then, 2.3 μL of calcein AM (Chemie Brunschwig, BIO80011-1) and 3.2 μL of Ethidium homodimer (EH, Chemie Brunschwig, BIO30002) dissolved in 10 mL of PBS were added into each well, and the plate was incubated for 20 min at 37 °C. At the end of the incubation time, the staining solution was aspirated, and 400 μL of PBS was added onto glass slides to prevent drying of the cells before microscopy. Imaging was carried out with an inverted Leica SP8 confocal microscope. Live/dead assay was performed in triplicate. DMEM with 5000 cells/well was used as control.

#### (c) Fluorescent Staining

Fibroblast cells were seeded on the sterile coverslips at the density of 7500 cells/well. Then, conditioned medium where hydrogels incubated for 1 and 2 weeks was added to each well and the well-plate (12-well) was incubated for 3 days under standard cell culture conditions. At the end of the incubation time, the medium was gently aspirated, and cells were fixed with 4% paraformaldehyde solution (Lucerna Chem AG, 15952) for 10 min. After removing the fixation solution and washing the cells with PBS two times, Alexa Fluor 568 phalloidin solution (1:400 in PBS, Invitrogen, A12380) was put into each well to stain the cytoskeleton of the cells. Then, the plate was covered with aluminum foil and incubated for 40 min at room temperature while shaking the plate gently. At the end of 40 min, the solution was aspirated, and each well was washed three times with PBS for 5 min. After the washing procedure, DAPI solution (1:10000, Thermo Fischer, 62248) was added to stain the nuclei of the cells, and the plate was incubated at room temperature under gentle shaking for 10 min. Next, samples were washed with PBS three times before imaging. Imaging of each slide was performed with an inverted Leica SP8 confocal microscope. Fluorescencent staining was performed in triplicates. DMEM with 7500 cells/well was used as control.

### Toxicity of the Degradation Product

Mouse embryonic fibroblast cells (NiH3T3) were used to evaluate the toxicity of the degraded products under enzymatic and oxidative conditions. Enzyme activity of the enzymatic degradation medium (lipase and lysozyme) was neutralized in an oven at 75 °C for 30 min. Since H_2_O_2_ is also harmful for cells, catalase enzyme was used to convert all H_2_O_2_ molecules to H_2_O and O_2_ by reacting 2 mg of catalase with H_2_O_2_ degradation medium for 5 min. After that, like other enzymatic solutions, catalase activity was also neutralized in an oven at 75 °C for 30 min. Then, all degradation solutions were sterile filtered under a laminar flow. Sterile solutions were diluted 1:9 (degradation medium:DMEM), put into 96 well-plates containing 1000 cells/well, and incubated for 1 day. After the incubation time, medium was aspirated from the well-plate, and 100 μL of 10% (v/v) PrestoBlue (A13261, Life Technologies) was put into each well and the plate was incubated for 30 min. After that, fluorescence was measured at 595 nm using a microplate reader (Wallac 1420 Victor2, PerkinElmer). The experiments were performed in triplicate using five replicates for each experiment. DMEM with 1000 cells/well was used as control.

### *Ex Vivo* Experiments

To mimic the extreme tracheomalacia condition, 4 to 5 cartilage rings were excised from rabbit tracheas with a scalpel, ensuring the inner tracheal mucosa remained undamaged. The distal end of the trachea was closed using surgical sutures to maintain an airtight environment. The flexible bronchoscope was then inserted into the trachea through the laryngeal inlet. The hydrogel patch (15 × 35 × 0.7 mm^3^) was prepared as described earlier. Then, the precursor solution was poured onto the tracheal surface, and the preformed hydrogel patch was wrapped around it, avoiding any air bubble formation. The second photopolymerization step was then performed using a portable torch for 5 min. Then, a negative pressure (−5 kPa, maximum physiological pressure of human) was applied to the trachea by suction setup (Medela Surgicals) and collapse behavior was observed and recorded by a flexible bronchoscope (Boston Scientific). Experiment was repeated 3 times, subjecting the trachea to 20 ± pressure cycles. To quantify the results, the open area of the airway was calculated using ImageJ 1.51 software (National Institute of Health, Bethesda, Maryland).

### Statistical Analysis

All data were reported as mean ± SEM. Statistical parameters of the data were presented in the corresponding section of the methods above. The OriginLab software (Northampton, MA) was used for statistical analysis of the data. One-way analysis of variance (ANOVA) with Tukey’s test was applied for data analysis. A level of *p < 0.05 was used to determine statistical significance.

## Supporting Information Available

Effect of monomer and cross-linker concentration on adhesion, brittleness observed upon addition of 3 wt % PEG-NHS, preparation steps for glass adhesion measurement, and preparation steps for free-standing adhesion measurement (PDF)
