# Supplementary material for: Enhancing Robustness of Adhesive Hydrogels through PEG-NHS Incorporation
Source: ACS Appl Mater Interfaces. 2023 Oct 23;15(43):50095–105. doi: 10.1021/acsami.3c13062 (PMC10623379; doi:10.1021/acsami.3c13062)
Supplement: Supplementary file 1 — am3c13062_si_001.pdf [file am3c13062_si_001.pdf]

SUPPORTING INFORMATION

Enhancing Robustness of Adhesive Hydrogels Through PEG-NHS Incorporation

Ece Uslu[1], Vijay Kumar Rana[1*], Yanheng Guo[1], Theofanis Stampoultzis[1], François Gorostidi[2], Kishore Sandu[2], Dominique P. Pioletti[1*]

[1] *Laboratory of Biomechanical Orthopaedics, Institute of Bioengineering, School of Engineering, EPFL, Lausanne 1015, Switzerland.*

[2] *Airway Sector, Médecine Hautement Spécialisée, Department of Otorhinolaryngology, University Hospital, CHUV, Lausanne 1011, Switzerland.*

[*] *Corresponding authors: Vijay Kumar Rana, Email: vijay.rana@epfl.ch, Dominique P. Pioletti, Email: dominique.pioletti@epfl.ch*

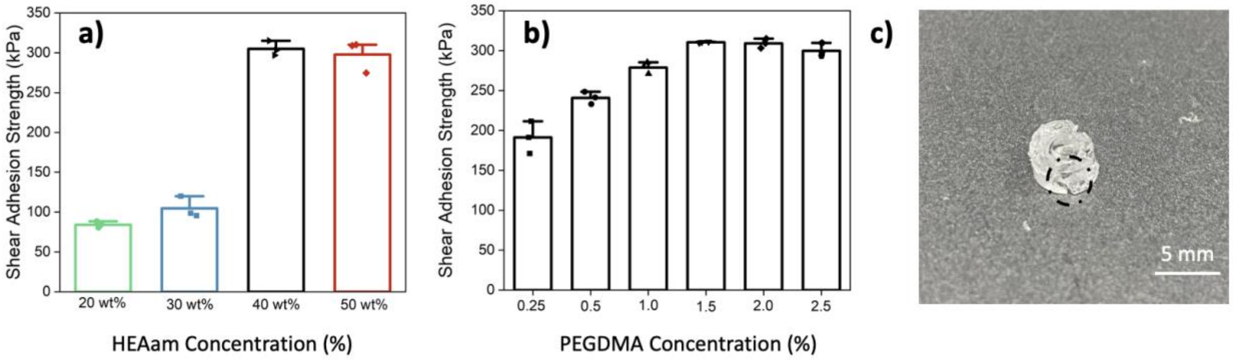

Figure S1. The effect of **a)** monomer (HEAam) and **b)** cross-linker (PEGDMA) concentration on adhesion strength of HEAam-based hydrogels on gelatin-coated glass surfaces. **c)** Brittleness observed upon addition of 3 wt% of PEG-NHS into HEAam polymer network. Data are represented as mean ± SEM.

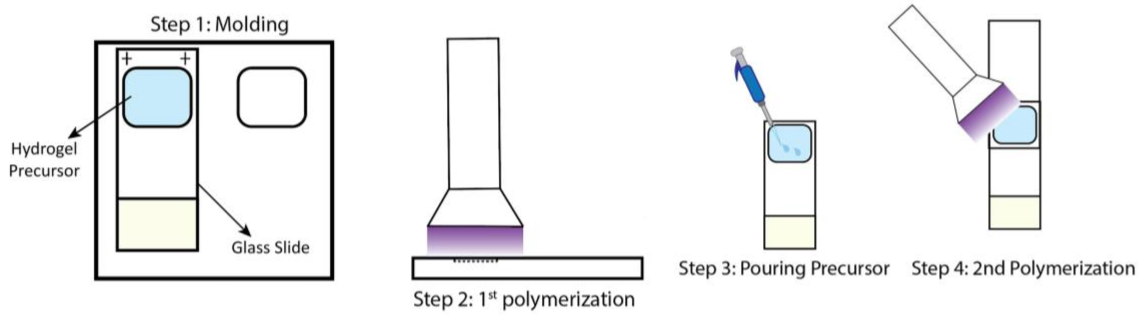

Figure S2: Preparation steps for glass adhesion measurement.

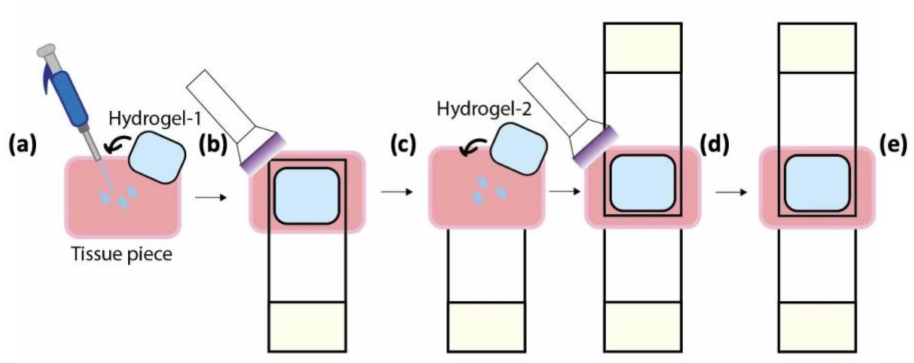

Figure S3: (a) 100 µL of precursor solution was poured onto heart, liver, lung, or kidney surfaces and then preformed adhesive hydrogel on the glass slide was placed, (b) second polymerization step was performed, (c-d) same procedure was repeated on the other side of the tissue surface and

(e) free-standing tissue with two preformed hydrogel was obtained to measure their adhesion performances.
